# Criteria and Guidelines for Human-Centered Work Design in a Digitally Transformed World of Work: Findings from a Formal Consensus Process

**DOI:** 10.3390/ijerph192315506

**Published:** 2022-11-23

**Authors:** Patricia Tegtmeier, Corinna Weber, Sabine Sommer, Anita Tisch, Sascha Wischniewski

**Affiliations:** 1 Federal Institute for Occupational Safety and Health, Friedrich-Henkel-Weg 1-25, 44149 Dortmund, Germany; 2 CWeber-Coaching, Wasserstraße 26, 46284 Dorsten, Germany; 3 Federal Institute for Occupational Safety and Health, Nöldnerstraße 40-42, 10317 Berlin, Germany

**Keywords:** future of work, occupational safety and health, guidelines, digitalization, nominal group technique, real-time Delphi

## Abstract

With the increasing digital transformation, work tasks are changing—in some cases, significantly. Our study addresses the question of whether the established criteria for work design are still sufficient or if they should get updated and additional criteria become necessary in the context of digitalization. In a multistage consensus process involving interdisciplinary groups of experts, we have identified specific criteria for the humane design of work in a world increasingly permeated by digitalized work tools. Starting with an expert workshop using a combined nominal group/focus group technique, followed by a real-time Delphi study, a content analysis and a five-stage peer comment process, we detected 13 criteria and 38 design guidelines for human-centered work in digital transformation. Mapping these with established criteria, it became apparent that some established criteria have experienced a new dynamic because of the digital transformation. For other criteria, a need for digitization-sensitive design is discernible. In addition, criteria have emerged whose necessity is rooted in the digital transformation. A diffusion and stronger interconnection of the various levels of the work system in connection with the digital transformation of work is apparent.

## 1. Introduction

The digital transformation of the world of work is a much-discussed topic, and as such has already been talked about in a wide variety of formats [1]. Technologies used for work range from robots to ubiquitous computing to big data. Following Grover and van Amelsvoortn [2], we consider the digital transformation of work as the change of work activities and organization as well as business processes through digital, databased technologies. This does not automatically lead to disruptive changes in work [3,4].

The Integration of technology into the workplace is a persistent theme in the design of work. One core of the current digital transformation is the interconnection of technologies and people. This enables a new level of globally networked work as well as more individualized and flexible work [5,6]. As a result, for example, greater flexibility can also lead to an increased interweaving of private everyday life and work [7,8]. New possibilities also emerge for work-integrated learning [9,10,11] or cognitive support for employees [4,12], which have implications not only for the tasks of individual employees, but also for the overall work system. The digital transformation of work thus forms an integral part of other sociocultural, political and ethical processes of change [13,14,15].

With the increasing digitization and networking of systems, the activities and tasks that people take on as part of their work are changing, sometimes significantly. Increasing automation means that a large number of occupational tasks are no longer required or are replaced by others [16,17,18]. The resulting change in tasks poses a key challenge for work design. In this context, the use of digital technologies at work does not automatically result in a load-optimized work design or a deterioration of working conditions [4,12,19]. It is therefore important to design technically feasible and potentially economically positive developments of the digital transformation with a view to the human being in the system of work, and from a societal perspective, being prospectively positive and human-centered [20,21,22,23]. However, it is still unclear what criteria should apply to the human-centered design of work in a world increasingly permeated by digitized work tools. Are established criteria in work science sufficient, or should they be updated and supplemented?

Ever-new developments of technological possibilities and their uses in work characterize the current scientific discourse [18,24]. In research projects in the field of occupational science, we see a tendency to focus on technology and its degree of innovation. Studies often look at prototypes in pilot applications. In a study on the importance of different technologies for future manufacturing, large differences emerge in the estimated importance of different technologies and the extent of their current active use in companies [25]. Additionally, other technologies are not yet developed and possible effects are correspondingly limited in their predictability [26]. This complicates the search for future-oriented occupational health and safety criteria for a humane design of work that will also hold up in the longer term [4,6,19,27].

Under similar conditions, consensus methods have been successfully used several times in other research areas to develop valid criteria and design recommendations [28,29,30,31]. By adopting this methodological approach, the present work contributes to answering the question concerning the criteria in a systematic way. Using a multistage consensus process involving interdisciplinary groups of experts, we have developed criteria and guidelines for the human-centered design of work in a digitized working world, validated the criteria and compared them with existing concepts.

Starting with a cursory look at established concepts of work design, we follow up with the methodical procedure for the development of the new criteria and design guidelines. The results of the process, including the criteria developed and the specific design guidelines, appear in the results section. We then elaborate on these extracted criteria classified in a model with respect to effects of the digital transformation in the discussion. Finally, in the summary, we point to both a concrete need for action and existing research gaps.

### Established Criteria for Human-Centered Design of Work Tasks

In particular for the design of work tasks, there are established concepts for the preservation of the health and performance of employees and the promotion of personality development [32,33,34]. A holistic approach to occupational safety and health (OSH) puts employees at the core of work design. Work can be considered humane if it does not harm the psychophysical health of workers and if at most it temporarily affects their psychosocial well-being, meets their needs and qualifications, enables influence on working conditions and work systems and contributes to the development of their personality [34]. Based on Rohmert, Hacker and Richter as well as Luczak and Volpert [35,36,37], central demands on the design for a performance and workload-optimized work design are therefore:

- Tasks should be free from harm;
- Workable;
- Free of impairment;
- Enhance learning and health.

**Figure 1:**
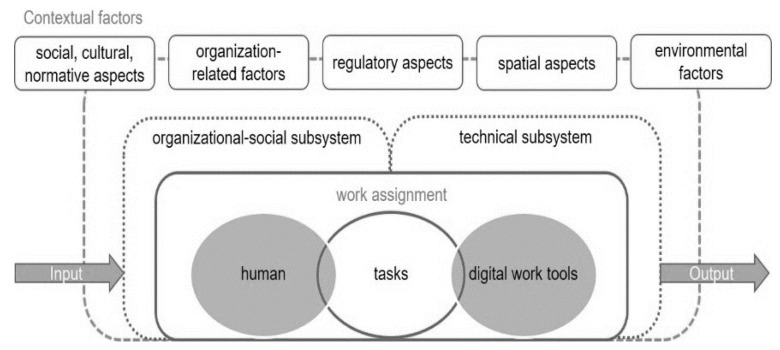
Model of a digital work system. Source: Weber et al. [41].

To this end, Ulich [34] describes seven criteria of a human-centered, personality- and health-promoting design of work tasks: Human-centered work tasks (1) emphasize completeness of a task, (2) offer skill variety and avoid one-sided strain, (3) allow for social interaction and (4) offer decision latitude (autonomy). They (5) offer time elasticity and counteract work intensification, (6) offer learning and development opportunities and (7) include meaningful activities. Work tasks do not stand in isolation. Embedded in the sociotechnical work system, they consequently connect employees with the technology and the organization [21,38,39]. Therefore, it is important to consider both human and technical resources when developing and implementing new technologies in a company. Environmental factors as well as social, cultural and normative expectations and experiences also affect the individual execution of the work task. Figure 1 shows the sociotechnical work system model composed of these factors.

Building on this model, in addition to criteria at the level of the specific work task, we therefore also address design features at the work system level and with a view to the contextual factors; and where available, we compare these with existing criteria. In addition, the human–organization interface is considered. The digital transformation enables a wide range of structures and forms in which work is organized. As a result, these roles and the associated responsibilities and duties of employers and employees are changing [40].

## 2. Materials and Methods

We developed the criteria and guidelines using a four-stage sequential mixed-methods design involving interdisciplinary groups of experts. Our approach included: (a) an expert workshop using a combined nominal group/focus group technique, (b) a real-time Delphi study, (c) a content analysis and (d) a five-iteration peer-commentary process.

### 2.1. Expert Workshop Using Combined Nominal Group and Focus Group Technique

A group of 15 experts took part in a two-day face-to-face workshop facilitated by an external moderator with the aim of initial identification of key trends and issues in the digitized world of work. The workshop ran under the Chatham House rule. Using nominal group technique [42,43] experts identified relevant topics on the future development of the world of work in the digital transformation, discussed these and grouped the generated topics in themes. Participants then worked in small focus groups on the themes in the context of digitization and safety and health at work. In a plenary session, experts grouped and completed the results and translated these into criteria for a human-centered work design. With the aim of creating a value-oriented image of the future, the experts agreed on different theses regarding risks and opportunities of future digital work environments as well as on associated design guidelines.

### 2.2. Real-Time Delphi

The theses and design guidelines were then the basis for a real-time Delphi [44,45] conducted between 7 October and 10 November 2019. We invited 95 national experts from the German Federal Institute for Occupational Safety and Health from various disciplines with research backgrounds in digital transformation and OSH to participate via email and a link to the web-based survey. Codes assigned by the external facilitator provided anonymity throughout the Delphi and after.

After a short introduction, including the criteria developed in the workshop, the panel members were asked to rate the theses belonging to a criterion according to agreement on a six-point scale (1 = not agree at all to 6 = fully agree) and the design notes created according to relevance for OSH (1 = totally irrelevant to 6 = very relevant). The order of processing was free. Participants could skip individual theses and design notes if they did not feel able to contribute. The panel members also had the opportunity to comment freely on the individual theses and design guidelines. Following the real-time Delphi method, the panel’s rating as well as all comments were immediately visible to the participants after each initial rating. We also asked the experts to comment on the relevance of the criteria underlying the theses and design notes in free text fields. In this way, they could indicate alternative names and still missing criteria. Additionally, they should identify the three most important criteria. Again, the survey showed the current ranking value per criterion after the first ranking.

Throughout the five-week survey period, the experts had the opportunity to return to their assessment via the provided link. They could adjust it in view to the current assessment of the entire panel or add further comments in reaction to ratings and comments of others. Since each user receives the average answers and reasons of their predecessors immediately after their own rating, even one-time users can immediately adjust ratings and respond to others’ comments. An important strength of this modified Delphi process is that it does not force consensus but instead seeks to identify where agreement exists while being explicit about where disagreement lies.

At the end of the survey period, we considered agreement with the formulated theses as well as the relevance of the design notes as given, if the resulting distribution was significantly left-skewed with a median greater than four. Conversely, we classified theses and design guidelines with a right-skewed distribution as rejected. The preference judgments on the criteria formed indicators for their relevance. We then qualitatively evaluated the free comments in a content analysis.

### 2.3. Content Analysis

Two members of our research team carried out a qualitative evaluation of the free comments in accordance with the content analysis according to [46]. This content-related analysis of the Delphi drew in particular on the following research questions:

- Which criteria did the experts consider particularly important?
- What did the panel participants feel was missing from the catalog of criteria?
- What did the experts identify as genuinely new effects of the digital transformation of work?
- Which criteria, theses, or design references were unclear or interpreted differently?
- Which criteria, theses, or design guidelines did the participants regard as irrelevant?

We were especially interested in explanations as well as in lines of argumentation for specific quantitative assessments. We also looked for references to literature or projects on individual criteria, theses, or design cues, as well as reported research ideas.

For this purpose, we applied a multistage procedure of category formation and coding. In a first step, both researchers coded the material available up to that point during the online commentary, using codes derived from the research questions, along the main themes. In a second step, we coded all the material again, differentiating and adding to the existing categories, in accordance with the process character of qualitative research [47]. To ensure the quality of the coding process, both researchers coded independently of each other using MAXQDA software and resolved any disagreements by discussion afterwards.

### 2.4. Five-Iteration Peer-Commentary Process

Based on the results of the content analysis as well as the ratings from the real-time Delphi, we revised the criteria and design guidelines identified in the workshop.

To do this, we first condensed the categories and subcategories of the content analysis and looked at interrelations between categories. In order to identify the specifics of the digital transformation of work, we compared the thus-defined criteria and design guidelines with established criteria for work design. We integrated the so-identified challenges to OSH into a model for working in the digitally transformed world of work. Then, we presented the reformulated, literature-enriched and supplemented criteria and design guidelines to an internal subsample of the online discussion for peer review in four iterations.

In order to finally assess the scientific applicability of the obtained results, the members of the Institute’s external scientific council received the formulated criteria and design guidelines. For this final peer comment, we asked researchers to critique the criteria and design guidance based on five guiding questions:

- How do you evaluate the overall findings?
- How do you view the criteria? Are they coherent and complete?
- How do you evaluate the classification of the criteria into the categories, established criteria, criteria with new dynamics and new criteria? Is this classification useful?
- How do you rate the design notes? Are they applicable in practice?
- Do you have any further comments on the guideline paper?

## 3. Results

### 3.1. Expert Workshop

The 15 experts identified through personal contacts of the project team had backgrounds in psychology (8), engineering (4) and social sciences, social policy, or sociology (3). The main research areas represented with regard to work design in the digital transformation included workplace, machine and operational safety; human factors and ergonomics; structural changes and work organization; working time and flexibilization, work and health; workplace intervention; and occupational safety and health structures and strategies.

These experts identified 14 criteria for human-centered work design with particular relevance in the digital transformation. For each criterion, the participants formulated two to three one-sentence theses for clarification and one design guideline for each thesis. This resulted in 36 theses and 36 associated design guidelines, depicted in Appendix A Table A1.

### 3.2. Real-Time Delphi

In the subsequent web-based, real-time Delphi, 68 of the invited experts (72%) participated in the ratings and the anonymous discussion. Due to the anonymity, no information is available on the specific research focus or sociodemographic data of these experts. Of the respondents, 38 (56%) used the option to log in multiple times. In the first possible week of the survey, 50 percent of the multiple users and 27 percent of the one-time users started their evaluation. Likewise, 27 percent of the one-time participants started the Delphi in the fifth, final week. In the group of multiple participants, this proportion was 11 percent.

Regarding the preference ranking of the three most important criteria, we found the following order:

1. Human decision-making authority (rated by 32.4% (N = 22) of the experts on rank one to three);
2. Transparency (rated by 25.0% (N = 17) of the experts on rank one to three);
3. Work densification (rated by 23.5% (N = 16) of the experts on rank one to three).

Scoring once, the criterion “Error Culture” is at the lower end of the preference ratings. Even though the expert panel rejected three individual theses, and some theses and design guidelines did not reach consensus, there was a consensus among the Delphi participants in favor of the 14 overarching criteria. None of the 14 criteria fell short of a place in the top three, indicating the significance of the individual criteria in the digital transformation with regard to OSH.

From the 36 initial theses, 19 reached consensus on the six-point scale with a median ≥ 5 and being significantly left-skewed. Of these, five reached the highest possible median of 6. Three were right-skewed with a median of three (twice) or of two (once). This implied a negative consensus for rejection. The remaining 14 reached no consensus, showing a symmetrical distribution with a median of four (Appendix A Table A2). Mean values ranged from 2.48 (SD = 1.32) for “A new image of man is required” to 5.78 (SD = 0.57) for “Option to capture and process a wide range of information”.

The design statements developed in the workshop consistently achieved high relevance ratings. In total, 31 reached consensus with a median ≥ 5 with a significant left-skewed distribution. The remaining five guidelines had a symmetrical distribution with a median of 4. Six design guidelines received the highest possible median of 6 and one had the lowest median of 4 (Appendix A Table A3). Mean values ranged from 4.05 (SD = 1.40) for “Anonymization of personal data in the medium run” to 5.54 (SD = 0.77) for “Visible employer responsibility for OSH in agile structures”.

### 3.3. Content Analysis

In addition to comments on the procedural approach, the real-time Delphi resulted in 1156 comments on content. Of these, 536 related to the theses on future developments of work, 37 to the proposed criteria for human-centered design of work in a digital transformed world of work and 583 to the design guidelines developed in the workshop. The minimum number of comments was 3 (thesis on “rising importance of remote management, virtual teams”) and the maximum number was 35 (thesis on “increasing risk of information overload”). We did not find any correlation between the average ratings and the number of comments.

Deductive codes, based on the research questions at the outset, concerned the criteria, theses and design guidelines (high importance, irrelevance, novelty, ambivalence, missing), as well as supplementary information (formulation cues, arguments and rationales for quantitative assessment and references to relevant literature and projects). New main codes inductively formed in the coding process were references to linkages between the material presented, societal and operational matters, comments on ethics and values in digital transformation and process-related comments. In addition, inductively generated subcodes differentiated the ex-ante established codes. The final coding scheme included 9 main codes with 25 sub codes (see Appendix A Table A4).

The in-depth evaluations of the content showed that the criterion of transparency encompasses various interrelated aspects. Here, the content analysis initially resulted in a differentiation into two aspects “technical system transparency” and “informational self-determination” due to different focal points. The comments further indicated that the criteria “technical system transparency” and “human decision-making authority” describe a common latent construct. Therefore, we combined both when the criteria were condensed. We present the relationships between the criteria revealed by the content analysis and the classification model in detail in the discussion section.

### 3.4. Five-Iteration Peer-Commentary Process

In a first step, we revised the criteria and design guidelines according to the results of the content analysis as well as the evaluations from the real-time Delphi. For seventeen of the presented theses, we were unable to establish a positive consensus based on the Delphi results. From the content analysis, one of the main criticisms of the theses was the lack of clarity due to their brevity and the lack of supporting evidence. In the final formulation of the criteria, we therefore dispensed with the short theses as explanatory material altogether. Instead, we illustrated the criteria with detailed texts based on the consensus theses as well as material from the initial workshop, and supported them with literature references. We then contrasted the formulated criteria with established work design criteria to identify specific aspects induced by digital transformation. This resulted in 13 criteria and the 31 design guidelines of human-centered work with positive consensus from the real-time Delphi. According to the results of the content analysis, we assigned eight of the design guidelines to other criteria.

The subsequent five stages of peer commentary served in revising the text. In the first four iterations, seven of the real-time Delphi experts participated in an open peer-review format. They represented research topics in workplace, machine and operational safety, human factors and ergonomics, neuroscience, working time and flexibilization, workplace interventions and occupational safety and health structures and strategies. The six researchers in the final fifth round, who reviewed the scientific applicability of the results obtained, expanded this spectrum of research topics, including, among others, economic aspects of occupational research, risk assessment and occupational, social and environmental medicine.

This process led to seven additional design guidelines. Furthermore, the peer commentary suggested that the criteria should reflect positive design demands. As a result, we renamed six criteria while retaining their content, and added wording to three others. The qualitative evaluation in the final stage of the comment process showed broad support for the guidelines. Three participants emphasized the need to validate the design guidelines in practice, for example, through case studies (see limitations).

### 3.5. Design Guidelines

In the following, we present the final thirteen criteria for human-centered work design in the context of digital transformation resulting from the overall process (Since we developed the criteria and design guidelines with a German-speaking audience in mind, many references are likewise in German).

#### 3.5.1. Holistic Work Design

The design feature of holism is realized according to [34] by a work task that contains “planning, executing and controlling elements” and enables employees to review their work results with regard to the work requirements. Thus, employees can assess the importance of their activity for the overall task and their own work progress.

The high importance of holistically designed work tasks does not initially change because of the digital transformation. Rather, it seems important that even in work processes involving digital technologies, e.g., as assistance systems, all employees retain planning, executing and controlling elements of their work.

On the one hand, the use of digital technologies harbors the risk that a fragmentation or segmentation of work tasks will lead to small-scale, monotonous work tasks for individual employees. This does not give employees the opportunity to recognize the importance of their activities for the task as a whole, leading to further undesirable human-related consequences [48,49].

On the other hand, within a digitized world of work, work tasks with increasingly highly complex cognitive demands on humans (e.g., in the area of machine learning) may arise [48]. For workers facing such work demands, it is important to ensure that the work activity also includes cognitive regulation demands at other levels (e.g., no conscious automated processes). The following design guidelines result from the above:

- The holistic nature of a work activity should be a central criterion when deciding on the allocation of labor between humans and technology in the production and service process.
- Ensuring holistic work tasks needs to begin during the design or development of a digital technology and it should guide decisions and actions during its implementation and evaluation in the work process.
- Adequate change of activities should be included in the job design.

#### 3.5.2. Diversity of Requirements

According to [34], diversity of requirements, together with the aspects of holism and meaningfulness, all form the core of the perceived importance of one’s own work. The decisive factor here is the option of using different knowledge, skills and abilities to perform a task. Furthermore, it is beneficial if physical and mental demands alternate and not only short-cycled activities occur. Challenges with realistic demands that are neither too simple (monotony and saturation) nor too complex are important here. This avoids one-sided stress (physical and cognitive) and promotes development.

The diversity of requirements at the level of the work task continues to be a relevant criterion for human-centered work design, which, however, is also becoming digitization-sensitive in some cases against the backdrop of the digital transformation of work.

On the one hand, automation and digitization can lead to a noticeable physical relief for those affected by technological change [50]. Even if physical work does not disappear in the course of the digital transformation, the proportion of jobs with a reduced degree of physically stressful task elements is increasing. Physical inactivity and prolonged sitting at work are associated with an increased risk of cardiovascular disease, obesity and diabetes [51,52]. If the workload shifts away from manual activities toward more cognitive requirements, this may result in an increasing proportion of work processes that involve little movement. At the same time, the use of mobile information and communication technologies, especially in flexible work contexts, can lead to increased physical stress for employees [7,53,54].

On the other hand, digital systems can process and provide information faster, more extensively and in more detail. For example, digital systems and networked systems can capture and manage more business and process data. In the interaction between people, digital work processes and work tools, the risk of human information overload arises due to the quantity and complexity of the data involved [50,55]. Among other things, these demands are associated with impairments in mental health [56]. At the same time, the use of suitable digital assistance systems and algorithms can support user-friendly information processing through appropriate preparation and presentation. This can also counteract mental stress and strain in the face of increasing data volumes and complexity [57]. The following design guidelines provide assistance in addressing these issues:

- Exercise should become an active part of a sedentary workday.
- Mobile workstations should also consider physical ergonomics.
- The amount and complexity of the information provided should be processable by humans.

#### 3.5.3. Time Elasticity

Time elasticity as a design feature of the work task protects against “inappropriate work compaction” [34]. Time buffers are therefore an important element in setting up work and time schedules.

The criterion of time elasticity continues to be a relevant criterion. Digital transformation can make work processes more efficient, as data and decision-making bases are available more quickly than before. Such digitized, more efficient work processes can result in an intensification of work for employees, leading to time and performance pressures. The process of introducing new technologies may itself also increase the intensity of work [7,58].

In order to minimize hazards to health and wellbeing due to excessive workloads, it is therefore necessary to design the amount of work and working hours accordingly for each employee. This results in the following design guideline:

- The ratio of work quantity and working time must balance each other properly.

#### 3.5.4. Opportunities for Interaction

In order to make the work task conducive to motivation, personality and health, according to [34], it should include opportunities for social interaction. Exchange with and social support by peers represent important resources that can help buffer the potential negative effects of work stressors [19].

The importance of this design criterion takes on particular relevance in the digital transformation, as the rapid increase in technical communication possibilities and the associated opportunities to work together at different locations in different time zones may be associated with different and new forms of interaction. Relationship building and trust are particular challenges of digitally mediated communication, since the rather spontaneous informal exchange that is important for this becomes more difficult [59,60].

The decision about whether interaction with colleagues should take place face to face or using a digital communication medium, or when to use which medium, should be made according to the purpose and content of the intended communication [61]. Not all communication content is equally suitable for all communication media. How well the communicators already know each other and how familiar they are with each other are also important [62]. Various communication options should therefore be applicable for the interaction of colleagues with each other. Accordingly, the following design guidelines arise:

- Digital work should include opportunities for direct and non-digitally mediated communication as well as collegial exchange.
- The choice of communication medium should be appropriate for the communication content.

#### 3.5.5. Appropriate Scope of Job Control

Job control comprises degrees of freedom in the processing of tasks, which manifest themselves in the scopes of action, design and decision making [34]. Scope of action includes the possibility to choose the procedure, the means of work and the time organization assigned to an individual or a team. Scope of design manifests itself in the diversity of the subactivities and the design options of a task, e.g., the sequence of different subactivities. Decision latitude refers to the extent of autonomy for the definition of tasks and activities or their delimitation.

The importance of adequate job control takes on a digitization-sensitive dynamic. On the one hand, technical systems offer the possibility of prescribing small-scale work steps associated with the risk of restrictions in scope of action and decision latitude. At the same time, the use of technical systems offers employees the opportunity to expand their scopes of action and design, for example by taking advantage of the possibilities of ubiquitous workflows [58]. Concurrently, expanded decision latitude and scopes of action and design can also lead to an intensification of work that is detrimental to health, possibly accompanied by a blurring of work boundaries due to constant accessibility, interruptions and information overload, as well as deadline and performance pressure [7,8,63,64,65]. The following design guidelines support reasonable job control:

- Degrees of freedom in task processing should be maintained, and where possible and individually desired, expanded through system design.
- It should be possible to limit one’s own responsibility for task processing.

#### 3.5.6. Work-Integrated Learning

Learning in the work process occurs, among other things, through the analysis of one’s own tasks. For a human-centered work design, it is important to promote learning or to create conditions favorable to learning. For example, tackling new or challenging tasks in the context of the work process can promote and expand existing knowledge and skills [66,67]. It is also important for the sustainable development of existing, and the acquisition of additional competencies, that employees are not under- or overchallenged [32]. Challenges depend not only on the task design, but also on the person performing the task and on the structural, cultural and social framework of the organization [11,66]. Appropriate learning support, e.g., by leaders, can additionally support work-integrated learning processes and counteract permanent under- or overload, e.g., by regularly reflecting on work and learning experiences and focusing on learning and development goals, not only on task performance [68].

Existing dimensions of work design conducive to learning retain their relevance in the digital transformation. In addition, the criterion of conduciveness to learning gains a digitization-sensitive dynamic, especially through digital work assistance systems. In digital work, technical, product-related and organizational innovation cycles are shortening. In this context, knowledge-intensive activities are increasing and work content is becoming more complex [69]. These require continuous development of adaptability, flexibility, professional self-efficacy and competence on the part of employees. Work-integrated learning is becoming increasingly important [11,70,71]. In contrast to formal learning (courses, seminars, etc.), informal learning can take place in a timely and workplace-oriented way. Compared to formally acquired knowledge, this immediate proximity to the work environment facilitates transfer to one’s own work context [72].

By providing, for example, context-sensitive information, digital learning and work assistance systems can contribute to supporting work-integrated learning in a meaningful way [73,74]. Particularly in light of the high diversity of modern work forces, digital learning media offer the potential to adapt to the individual requirements of employees [69,75]. This requires employees to have the appropriate digital skills, which they may first have to learn. In addition, the use of digital learning and work assistance systems should avoid de-skilling through the specification of small-scale work steps and preserve the holistic nature of the work task [48].

The use of digital technologies, including the learning and assistance systems mentioned above, can also lead to an increased sense of performance monitoring, as well as increased information overload and complexity [55]. The challenge lies in a balanced design of support solutions that challenge employees without overtaxing them, while not counteracting the actual learning process through too much support [74,75,76]. In this context, it is particularly important for manufacturers to adopt a user perspective as early as the development process. For example, usability studies with potential users can point out problems regarding complexity and the amount of information. A participatory organizational introduction of new technologies also can help to identify users’ expectations and difficulties in the concrete work context. That way, it is possible to identify and implement the need for action and additional training at an early stage [77].

Employees themselves can make a significant contribution to the design of work. Through their daily dealings with the work process, they have (partly implicit) experiential knowledge both about problems that arise and about possible solutions. Incorporating this knowledge into processes of systematic preventive work design is one of the challenges of the digital transformation of work [76]. The following design guidelines contribute to work-integrated learning:

- The integration of technical innovations into the work process should take into account and preserve the value of human experiential knowledge.
- Digital assistance systems should support employees in their activities where necessary, but continue to provide incentives for cognitive engagement, learning and development.
- Work design should take into account opportunities offered by digital technologies for work-integrated learning.

#### 3.5.7. Human-Centered Flexibility

The digital transformation affects the level of the organizational–social subsystem of work by providing a high degree of time- and location-flexible forms of work design. Here, the need for a new criterion of human-centered flexibility becomes apparent.

For many employees, the possibilities of flexible working represent a great opportunity for an improved work–life balance [78,79]. Options for location-flexible working can also offer relief to commuters [80]. However, these freedoms for employees often go along with high demands and expectations on the part of companies. This can lead to strong pressure to meet deadlines and perform. Working hours are extended and important rest periods for recovery are not respected [81]. Similarly, extended accessibility via mobile digital communications can blur the boundaries between work and private life [7,8,65]. The demands for self-organization that often go hand in hand with flexibility offers can overtax employees [82]. The design of framework conditions such as personnel selection or clear rules for interaction can have a positive effect here [83,84].

Furthermore, if employees in digital work systems only meet sporadically in workplaces or meetings and instead communicate primarily via electronic media, there is a risk that supervisors as well as colleagues will not perceive signals of interested self-endangerment [85].

Taking into account the following design considerations, flexible working can be human-centered and health-promoting:

- The opportunities offered by digital technologies for balancing work and family life should be seized.
- Even in flexible forms of work, employees should have a right to be unavailable outside their agreed working hours.
- To protect against motivated self-endangerment, managers should be able to assess the stress levels of their employees regardless of where they work and take preventive action.

#### 3.5.8. Fair Evaluation Processes

Digital transformation not only enables location- and time-flexible working, but also the collection of large amounts of data, which are useful for decision-making at various levels in companies. At the same time, however, the use of the data thus obtained and aggregated allows for opportunities for performance monitoring and control [48,86]. In addition to the existing possibilities for using such data to select and/or evaluate employees by human players in organizations, it is also increasingly possible to use autonomous algorithmic decision systems for this purpose. If such systems are to make decisions, the underlying data must first be suitable for achieving meaningful and fair judgments. In order to determine whether further aspects previously unconsidered require attention, human judgment is still required at the current state of technology [87,88].

Furthermore, especially in the field of new forms of work such as crowd or platform work, the use of digital assessment systems as a basis for job assignment and thus for task allocation can be observed to an increasing extent. This can have a significant impact on the long-term employment opportunities of the evaluated, as their job situation is directly dependent on the results of the evaluation systems [89,90]. Of particular importance are the weight and duration of individual evaluations on the evaluation system. Continuous evaluations by third parties, e.g., customers, also can give rise to massive uncertainties on the part of the employees, which can have a detrimental effect on their health and well-being.

The following design guidelines result from the above:

- Individual performance monitoring should not be fully automated.
- Humans should regularly check decisions made by digital and especially self-learning systems for plausibility and fairness.
- To protect employees from the negative consequences of third-party performance evaluations, the design of digital systems should be robust against outliers and grant a “right to be forgotten”.
- Employees should have the possibility to independently save and use built-up digital assessment data.

#### 3.5.9. Human Decision-Making Authority and Technical System Transparency

Algorithms can compute optimized solutions to problems from existing data, and thus may be able to contribute to faster, more efficient and more comprehensive solutions and decisions than humans can in the same amount of time [91]. However, due to the associated high complexity of technical systems, their transparency can get lost for employees [19,88,92]. This can have an unfavorable effect on human–system interaction in the context of work. With the increasing use of such systems in the work context, the need to address this challenge also increases.

A lack of transparency can have an unfavorable impact on several areas of human decision-making authority [93,94]. For example, it may result in a loss of expertise, so employees cannot make appropriate responses when critical incidents occur [76,95,96]. In addition, in order to prevent operating errors, as well as overconfidence in the technology, a clear understanding of the basic functioning and objective possibilities of the technology used is required.

Especially in situations where difficulties or errors arise in the work process, a clear attribution of responsibilities is necessary. In digitized work processes, the integration of technical systems creates the particular challenge of understanding these responsibilities, since technical systems cannot assume responsibility themselves [97]. Innovative systems are increasingly capable of providing human-like services (e.g., writing texts, processing telephone inquiries). This can make it difficult for employees to recognize to what extent an interaction, e.g., by telephone, is taking place with a human or a machine, or which products have been created autonomously by an algorithm [93]. The following design guidelines result from the above:

- Humans should be able to retain decision-making authority and control over the functions of technical systems.
- The decision of algorithms in work processes should be verifiable in all functional areas by suitable specialist personnel.
- Given the condition of technical transparency, there is a clear, communicated assignment of responsibilities for procedures and decision making.
- Interaction with autonomous systems should be immediately apparent to users.

#### 3.5.10. Reliability of Technology

The reliability of the technology plays a central role at the level of the work subsystems as well as in the interaction with the work task. In connection with the digital transformation, new dynamics and challenges for work design are developing—in some cases, very rapidly.

It is likely that digital technologies will increasingly support employees in their work processes, or partially or completely replace them. The freedom from interference and errors of systems such as video systems, VPN tunnels, or network coverage will become a condition for avoiding misuse and/or endangerment of employees interacting with the technologies [55,98].

Increasing networking through digital technologies is observable. Ensuring the functional safety of increasingly complex systems, facilities and machines and making them accessible for testing presents new challenges and requires the further development of measures and methods. An additional problem arises from possible manipulations and misapplications due to networking [99,100]. A particular focus is on attack security in combination with functional security. Work design in this area of potential conflict can therefore only take place on an interdisciplinary basis with the involvement of cyber security experts.

As learning systems proliferate, ensuring relevant training data is required [92]. While classically only the algorithm needs testing, in learning systems a faulty training database can also compromise technique reliability [94]. This is a current research topic for which no established procedures have yet emerged. The following design guidelines result from the above:

- Work support technology should have a predefined reliability and safety level assessable by means of suitable analysis methods.
- Security measures to protect against tampering should have a predefined reliability at all times and be accessible to an appropriate risk assessment that takes into account not only the type of digital application but also the context of use, such as the work task and requirements.
- For learning systems, testing mechanisms should be available to allow for verification and validation of the underlying algorithm.
- For learning systems, checking mechanisms should be in place to ensure the plausibility of data and correct results.
- Defined quality criteria exist for training data that allow for later use of the learned model with a defined reliability.
- Accompanying monitoring over a defined period after commissioning of the learning systems should occur in order to be able to analyze, evaluate, and if necessary, improve reliability in the context of use.

#### 3.5.11. Human-Centered Use of Technical Innovations

Closely following the criterion of meaningfulness formulated by [34] with reference to the work task and in combination with an extended perspective beyond the task level of the work system is the criterion of “human-centered use of technical innovations”. The design of a work task counts as meaningful if the completion of the work task can convey a feeling of operational and/or social benefit [34]. With reference to the concept of coherence, a sense of meaning at work occurs when employees can establish a correspondence between their own values, goals and ideas of benefit with those of the company or those prevailing in their workplace [101,102].

In new forms of control and distribution of work, such as crowd and platform work, it is possible to outsource isolated work steps. Particularly in cases where a fragmentation of originally complex activities comes with de-skilling, limited opportunities for action and increased control, the sense of meaning can also be impaired. Moreover, employees may experience the introduction of technologies in the context of digital transformation as restructuring. Such an experience of restructuring can jeopardize the sense of meaning at work if the perceived corporate values and norms no longer match one’s own, due to the change processes.

The decision to use digital technologies should not be made for the sake of using the technology, but based on its usability, taking into account economic and social influencing factors, so that employees can attribute meaning to their work activity as well as to the choice of work equipment at any time.

Meaningfulness through a human-centered use of technical innovations is effective not only at the task level of work, but also at the contextual level of the work system, which, among others, takes into account social, cultural and normative influences. Thus, meaningfulness can also occur for employees if they can contribute to a societal or social benefit through their work that corresponds to their values and has an identity-forming effect [34,103]. Accordingly, the social benefits of technical innovations should receive consideration.

At the level of organization-related contextual factors, digital technologies should be implemented with regard to their potential for individual work design. Digital technologies have the potential to enable inclusion and promote diversity, for example, by reducing barriers and inequalities in the workplace [104]. Digital technologies facilitate the consideration of individual performance requirements and the needs of different employees in the design of work. The following design guidelines result from the above:

- The use of technical innovations in the work context should enable meaningful task processing for people.
- The use of technical innovations in the work context should involve an assessment of its benefits for society.
- The individual adaptability of digital technologies for employees should receive consideration in the technical design and organizational use.

#### 3.5.12. Inclusiveness, Consideration of Individuality and Diversity

With the use of innovative technologies, the opportunities for differential work design increase [19]. Linked to this, new opportunities for participation arise at the level of contextual factors in the area of social and cultural aspects, which are just as much of interest in terms of social policy as they are in terms of employment policy [104].

Thus, digital assistance systems and technological aids can help compensate for physical impairments, especially those caused by physical and sensory disabilities [75,105,106,107]. Forms of work that decouple working from a specific location, such as cloud and crowd working and telecommuting, also offer improved employment opportunities for people with mobility impairments [105,108]. However, complete decoupling from the workplace can exclude from work-related communication, team meetings and informal contacts. It is important to consider the resulting challenges accordingly, for example, with regard to changing leadership requirements [109].

The use of digital assistance systems can also offer opportunities for individual work design, for example, in the course of the day or along the employment biography, and thus open up expanded possibilities for orientation to individual needs and requirements in the interaction between work and other areas of life [18].

In the course of increased use of digital technologies and greater networking of systems, work processes are also becoming more demanding and complex and require correspondingly higher skills and abilities on the part of employees. This may worsen employment opportunities, especially for people with cognitive impairments [105]. Here, there is a close link with the criteria of diversity of requirements and work-integrated learning. If skills and abilities are supported and further developed using cognitive and tutorial assistance systems, there are also opportunities for this group of people to increase their employability in the longer term [18]. The following design guideline results from the above:

- The opportunities that new technologies offer for work design should also provide for inclusion and diversity.

#### 3.5.13. Clear Responsibilities for Occupational Safety and Health

Organizational, regulatory and spatial aspects of the work system influence the effectiveness of OSH. The use of digital technologies has a direct connection with the organization of work and influences organizational and social structures and processes that are relevant to occupational safety and health. The growing networking and software support of production and business processes enables a wide range of flexible forms of work, i.e., independent of fixed locations, standardized working hours and stable organizational structures. Rules and procedures in OSH and the associated rights and obligations of employers and employees have so far tended to be oriented toward a world with fixed, tractable and familiar workplaces and describable work situations. In the course of the digital transformation, the question of responsibility in occupational safety and health is taking on a new dynamic.

The rapidly changing market processes, associated with digital transformation, create a need for companies to adapt flexibly to changes that are fraught with uncertainty. In order to be able to react quickly and flexibly to market adjustments, many companies are changing their structures and processes. Alternative flexible forms of organization are discussed in this context, with the keywords of participation, self-organization, decentralization or flat hierarchies and agility [110].

Important aspects of such organizational forms include the autonomy of employees and the self-organization of teams. To benefit from innovation and productivity gains, employees must take responsibility for development steps within their own work. It enables teams to act faster and respond more flexibly to changes at short notice. This is associated with greater demands on leadership, which is detached from hierarchies and increasingly takes place at eye level [76,109]. From an occupational safety and health safety perspective, there is a risk that a diffusion of responsibility may arise, particularly in agile teams, as to who is responsible for compliance with the employer’s duty of care in the team.

The effectiveness of occupational safety and health may also suffer from the fact that many activities no longer necessarily require a specific work location or fixed working hours due to digital technologies. Responsibility for OSH and the duty of care for their employees continues to lie with managers. The physical distance makes it more difficult for them to identify hazards to employees and to monitor the implementation of necessary protective measures, and so requires new tools and procedures.

In addition to changing intracompany organizational structures, digital technologies also support a wide range of networked work structures in which workers engage work processes via supply chains and or platform-mediated work [6]. In these forms of collaboration, it must be clarified which of the participants have system and process step responsibility, and to what extent, and thus hazards associated with the work in each case (hazard assessments) must also be identified and assessed as well as the necessary health and safety measures implemented and check their effectiveness.

When using technical work equipment and technical systems, the distinction between manufacturer and operator becomes increasingly blurred. Based on the systematic processing of data and information about the work process, work equipment and machines can assume autonomous control functions in real time and reconfigure themselves. For this interface between product safety and OSH, questions arise about responsibility for risk assessment and hazard evaluation. The following design guidelines result from the above:

- Particularly in agile forms of work, the employer should take responsibility for the safety and health of employees and make everyone involved aware of this.
- It is vital to implement OSH structures effectively even in the changed forms of work and employment.
- Managers should also exercise their responsibility for care when leading in spatially and temporally distributed forms of work.

## 4. Discussion

This study aimed to develop criteria and guidelines for the humane design of work in a digitized working world. Starting with an initial nominal group/focus group method, we validated the thus-generated criteria using a real-time Delphi and compared them with existing concepts (content analysis and five-stage peer commentary).

### 4.1. Research Methods

The multistage process proved to be very suitable for developing the criteria and design guidelines with a large group of OSH experts in terms of both breadth and depth of content. By drawing up the initial workshop with a broadly diversified group of researchers, a wide range of research knowledge on the future development of the digital transformation in the world of work emerged right from the start. The use of the nominal group technique facilitated an equal presentation of relevant topics with subsequent discussion and evaluation. The focus group technique enabled a more in-depth elaboration of the identified topics and an initial formulation of design guidelines. Due to the scope and prospective nature of the guideline to be developed, we felt that this method was significantly more efficient than the alternative of a preliminary review. The fact that the Delphi panelists confirmed the criteria as well as the majority of the established design guidelines, and at the same time did not point out any missing topics, increases our confidence in the chosen approach.

Given that a higher number of items leads to a significantly lower response rate in the second round of a classical Delphi [111], we see a clear advantage of using the real-time Delphi. The real-time Delphi with 36 theses and 36 design guidelines, for the 14 formulated criteria, enabled an evaluation of a comparatively high number of items, including the collection of open comments.

The qualitative comments from the real-time Delphi were very valuable for refining the criteria, while at the same time improving their comprehensibility. In particular, the criticism that the presented theses were not clear enough due to their brevity and lack of evidence convinced us to illustrate the criteria with background information and to provide literature references. We consider the subsequent iterative peer review process to be particularly important to ensure transparency in the development of the newly formulated criteria and design guidelines.

### 4.2. Criteria and Guidelines

We set out to answer the question of whether the established criteria for work design are sufficient for the digital transformation of work or whether they require updating and supplementing. The content analysis of the comments was very valuable for comparing the criteria generated in the consensus process with the already established criteria. Thus, using this multi-stage consensus methods design, involving interdisciplinary groups of experts, we also derived five central findings from the process.

**Figure 2:**
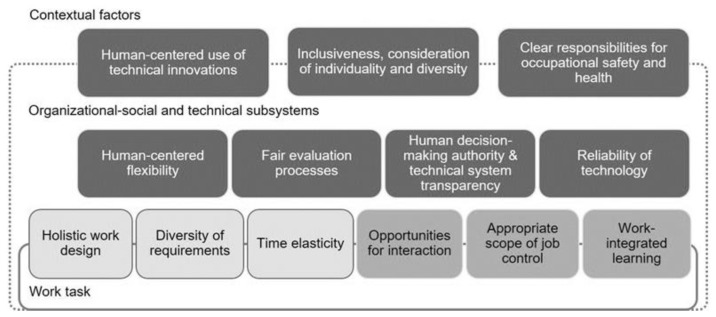
Criteria for human-centered work design in the digitized world of work. The lightest shade of grey indicates established criteria with confirmed relevance, the middle shade of grey indicates criteria that are sensitive to digitization and dark grey indicates criteria whose necessity only arises because of the digital transformation. Source: Weber et al. [41].

A large number of the criteria for a human-centered work design in the digital transformation relate to already-established criteria of human-centered work design (Figure 2). This especially applies to the criteria of holistic work design, diversity of requirements and time elasticity, confirming their continued relevance. For example, tasks performed with digital tools should include planning, execution and control elements, as well as activity changes. They should include meaningful activities and not be demanding in a one-sided way. Likewise, the need to fit the time available for completing a task applies regardless of whether the tasks are nondigital or digitally mediated. However, these three criteria already indicate a new dynamic due to advancing digitization. This reflects, for instance, the discussion about an increasing information overload due to the spread of digital ICT [112,113]. The demand for a holistic work design, in which more than residual activities remain with the employees, is also gaining new momentum, sometimes having different effects on different groups of employees [114], while the risk of physical inactivity at work, which has been observed for some time, is being considered in new work environments [115,116].

We have also identified a number of digitization-sensitive criteria characterized by a new quality of challenges. The risks and potentials of digital transformation lie in close juxtaposition to these criteria, resulting in a need for digitization-specific forms of design. Thus, social interactions happen in new, digitally mediated ways. While this can create opportunities for interaction in hybrid work, it may also negatively affect, for example, teambuilding [117]. The negotiation of decision making and operational latitude occurs not only between humans, but also in interaction with algorithms and artificial intelligence [118]. Likewise, the frequently rapidly changing use of digital work tools requires and enables changed forms of learning at work [119].

We were able to pinpoint criteria for human-centered work design that are only becoming necessary due to of the current digital transformation. Four of these criteria are at the level of organizational–social and technical subsystems. The aspect of human-centered flexibility with its opportunities and challenges appears in a wide variety of studies [120,121,122,123]. Regarding the criterion of fair evaluation processes, current research on chatbots, for example, indicate that they can facilitate recruitment processes. However, to make these evaluation processes fair and appropriate, recruiters need to pay a lot of attention to pre-defining their choices for the chatbots [124]. Looking at emerging human–artificial intelligence collaboration highlights, among others, the need for human decision-making authority and technical system transparency and reliability of technology [125]. Three further identified criteria refer on a superordinate level to the overall system of work specifically addressing aspects of social responsibility in work design. On the topic of inclusion/consideration of individuality and diversity, current studies address the opportunities and challenges of digital transformation for specific groups of employees [126,127,128]. Under the headings of sustainable and ethical organization [22,129,130], a wide variety of recent studies present contents that we have identified under the criterion of human-centered use of technical innovations. On the criterion of demand for a clear responsibility for occupational safety and health, research points to the problem of unclear categorizations of employers, employees and self-employed with regard to, e.g., platform work [131].

In addition to a change in the dynamics of individual criteria, we perceive a diffusion of system boundaries in the general picture. Assigning the individual criteria to different levels of the work system increasingly appears to be too limited. Even established criteria, previously primarily directed at the design of work tasks, often already gain design significance at the system level on the boundary between the technical and the organizational subsystem. For example, opportunities for social interaction can arise not only through the specific work task itself, but also through agile forms of organization or the use of digital systems, such as chat programs and virtual meeting rooms [132]. The task allocation between human and robot influences the direct task level in criterion holistic work design via the criterion of human decision-making authority and technical system transparency, and trigger discussions about an optimal balance between technology and society [133]. The evaluation of the open comments from the real-time Delphi also indicates a possible shift in the relevance of individual criteria for work design. Asked about their assessment of the most important aspects of work design in the digital transformation, the participants attributed the greatest importance to the aspect of human decision-making authority. The topics “transparency”, “work densification (subsequently renamed time flexibility)” and “technology reliability” followed closely behind. Since, according to the comments, the criteria “technical system transparency” and “human decision-making authority” describe a common latent construct, they appear as a common criterion in Figure 2. Thus, two of the criteria voted among the top three are at the subsystem level, highlighting the importance of work design at the subsystem level.

Finally, the content analysis of the Delphi comments shows that individual criteria, identified as significant, can also contradict each other. This illustrates, for example, controversial comments on the connections between technical system transparency and fair evaluation systems, as well as time elasticity. While technical system transparency is seen as an important basis for informational self-determination, it can, at the same time, contribute to information overload and work intensification, and thus impair time elasticity, since it can increase both the amount and the complexity of information that employees have to deal with [124]. Employees using unofficial workarounds to deal with inadequate information systems may experience positive effects with regard to the scope of job control as well as time elasticity. At the same time, however, these workarounds can potentially have a detrimental effect on the reliability of technology in the organization [134]. This interweaving of positive and negative effects on work design is a particular challenge in the digital transformation for occupational safety and health.

### 4.3. Implications

Our study looks at the digital transformation of work from a specific OSH perspective. In order to reflect the potential of digitization for a positive development of the world of work, the focus in developing the criteria deliberately lies on aspirational ideals. The 13 criteria take into account both technologies already used in everyday work and prognostic components. The proposed criteria cover a wide range of different technological developments and at the same time go beyond the assessment of individual specific technologies. This broad perspective on the digital transformation of work also allows the interlocking of the criteria with each other to emerge clearly. The three high-level criteria in particular also point to the social significance of work design in the new work reality. Validated by researchers from interdisciplinary backgrounds, these recommendations provide a good basis for further research.

Despite pending empirical testing in the field, our recommendations have also emerged with implementation by practitioners in mind. It is precisely with the 38 design guidelines, identified in the consensus process, that we hope to provide an initial implementation aid for those involved in occupational safety and health settings.

## 5. Limitations and Recommendations for Future Research

There are some limitations related to the process of generating the criteria. First, in the real-time Delphi format chosen, individuals who participated in the assessment and commentary only once at the beginning of the survey period can contribute little to iterative consensus building [45]. They also have less basis for reflecting on their own assessment. On the other hand, in our Delphi, half of the multiple participants started in week one of the survey and only a quarter of the one-time participants. Thus, from week two at the latest, a good data basis for the evaluations was available for all of them. In addition, the free text comments reveal a lively anonymous exchange of ideas and reasons for one’s own assessment, even among those who participated only once. We are therefore confident that we have also generated reliable assessments with this roundless procedure.

Nevertheless, it might be a good idea for future studies to send e-mail reminders to participants halfway through the time allotted for a real-time Delphi to encourage one-time participants to submit follow-up assessments.

Second, given the focus on national OSH implication, the samples that the study is based on are purely German. As a result, the design guidelines obtained also reflects a view aligned with the German occupational safety and health system and the work science approaches represented here. This limits generalization to other countries.

Further research is needed to formulate non-country-specific criteria here, if possible. With the criteria presented here, we hope to contribute to further research around the design of safety and health in digital transformation in general. Furthermore, we encourage other scholars to continue exploring the interactions of design criteria in the digital transformed work as well as the diffusion of boundaries in the system of work.

Third, the work design criteria generated are the result of a purely scientific consensus process. They have been formulated to support the development of OSH scenarios in the near future and thus to contribute prospectively to the design of work in the future. It is therefore possible that some of the formulated design guidelines are more applicable in practice as others.

Hence, further research is desirable that examines the implementation of the criteria in company-related workplaces, e.g., through case studies.

## 6. Conclusions

The digital transformation permeates all areas of work and acts as a driver of comprehensive changes. These include both diverse opportunities and risks affecting the system of work. A prospective, positive and design-oriented approach based on ergonomic findings and must focus on people in the digitized system of work. To this end, we require clear criteria for a human-centered design of work increasingly permeated by digitalized work tools.

In a multistage consensus process, we identified such criteria for human-centered work design in the digital transformation and mapped them against established findings on design. In addition to criteria that have experienced a new dynamic because of the digital transformation, we found criteria for which a need for digitization-sensitive design has emerged. In addition, criteria have arisen whose necessity is rooted in the digital transformation. Particularly noteworthy is a diffusion and greater interdependence of the different levels of the work system in connection with the digital transformation of work.

The criteria developed here and the concrete design guidelines provide initial guidelines for human-centered work design in digitized work. This will enable a prospective development of occupational safety and health in order to make tomorrow’s work not only more efficient and productive, but also to take advantage of the opportunities offered by the digital transformation in terms of safe, healthy and good work.

## Data Availability

Data supporting reported results are available in anonymized form in German on request only at the premises of the BAuA due to privacy restrictions.

## Appendix A

**Table A1:** Criteria, theses and design notes derived from the expert workshop *.

Criterion	Theses	Design Guideline
Work Intensification	In the digital transformation, the risk of information overload increases and the complexity of the information to be processed increases.	The amount and complexity of (digital) information should be processable for humans.
	The digital transformation is contributing to an increasing workload.	The ratio of work quantity and working time must balance each other properly. If the work intensity is high, correspondingly more recovery time should be available.
Decision Latitude	The use of digital technologies contributes to a polarization of the scope of activities: Some employees seem to be gaining more scope for activity, while others are facing increasing restrictions.	Degrees of freedom in task processing should be preserved, and where possible and beneficial, expanded through system design.
	If digital technologies support the processing of the work task in substeps, this results in restrictions on the degree of temporal freedom in the task execution.	The degree of system support must be determinable by the human.
	The use of digital technologies opens up scope for more personal responsibility (e.g., in location-flexible working).	Individual responsibility in the processing of tasks needs limits; organizational responsibility must occur in an appropriate way.
Technology reliability	Digitally networked systems carry the risk of attack or manipulation.	The use of digital technologies must have a reliability appropriate to the application, protected against manipulation at all times, especially by third parties.
	In the future, autonomous systems will make complex decisions without human intervention.	The decision of machine algorithms (AI) in technical processes must be safe and verifiable in all functional areas by suitable specialist personnel.
	Complex and networked technical systems are prone to errors.	Work support technology should be available without disruption.
Social interaction and support	Communication between people is increasingly taking place digitally.	Digital work must adhere to opportunities for direct and non-digitally mediated communication.
	Social support and collegial exchange are also important resources in the digital transformation.	Digital work must promote collegial exchange and social support.
Technical and social innovation	In the digital transformation, human experiential knowledge receives insufficient attention.	Technical innovations must harness human experiential knowledge.
	The use of technologies is driven by the technical possibilities.	All human criteria are imperative for the design and evaluation of technical innovations and their application.
	The increasing digitization of work systems requires a new idea of man (beyond “complex man” or “virtual man”).	Within this idea of man, the human ability to learn has an independent merit and a correspondingly high value.
Transparency	It will often no longer be apparent to humans whether they are interacting with another human or a machine, or which information and products exclusively algorithms create.	Interaction with, as well as the result of, autonomous systems must be immediately recognizable to users.
	Digital transformation enables the collection and processing of a wide range of information.	Functions of technical systems must be transparent to the user at all times with regard to data use and decision making.
Human decision-making authority	In the future, algorithms and self-learning systems will be able to make decisions faster and with the inclusion of a wide range of information.	Humans retain decision-making authority over both the functions of a technical system and how to deal with the results.
	Algorithms and self-learning systems can only make decisions based on the information available to them.	If system data are relevant for decision making, humans must check it regularly for plausibility and fairness.
Organizational change	Digital transformation is contributing to flatter hierarchies and agile working.	Even in agile organizational structures and processes and flat hierarchies, employers must take responsibility for the safety and health of employees and communicate this clearly.
	The digital transformation is giving rise to a large number of new forms of work and employment beyond the workplace (keyword: platform economy).	OSH structures also experience effective implementation in the new diverse forms of work and employment.
	In the course of digital transformation, management from a distance/of virtual teams is gaining in importance	Even in spatially and temporally distributed forms of work, managers should be able to fulfill their responsibility for caring satisfactorily for themselves and their employees.
Inclusion, diversity, individuality	Digital technologies make it possible to take individual needs and performance requirements into account when designing work.	Users can adapt digital technologies themselves inter- and intra-individually; the competencies required for this must be ensured in order to achieve positive, health-related effects.
	Not all people are able or have the opportunities to keep up with technological progress due to their individual situation.	Digital technologies should come into play with the support of disadvantaged and low-ability people in mind; no groups of people should experience exclusion.
	Digital technologies, through their customizability, enable learning-enhancing experiences for a wide range of employees.	Digital technologies aim to support physically or mentally impaired individuals (performance-disabled employees) to increase their skills and employability.
Fostering learning	Digital technologies contribute to specialization and provide work instructions in a small-scale manner (re-Taylorization).	An implementation of digital (assistance) systems/technologies must maintain and promote sufficient/multiple learning opportunities at work.
	In the digital transformation, innovation cycles are shortening and “change” is becoming the norm.	In (prospective) work design, opportunities for work-integrated (informal) learning must receive special (intensive) consideration.
Dissolution of boundaries	In the digital transformation, the risk of unrestricted working hours and constant availability is increasing.	Working hours and availability for work must be limited.
	In the course of the digital transformation, more and more employees will work on a mobile basis: this will make them and their stress more “invisible” to their colleagues and superiors.	In order to counteract interested self-endangerment, measures are necessary to make the stress of colleagues working on the move visible.
	In the course of the digital transformation, the boundary between work and private life is blurring.	Digital communication should promote work–life balance—without extending working hours.
Error culture	The fact that technical systems can be faulty but do not assume responsibility is gaining importance in the digital transformation.	Assuming technical transparency, the assignment and communication of process and decision-making responsibility must be unambiguous.
	Digital evaluation systems are making their way into the world of work.	Algorithms convert personal data into an anonymized form for medium-term storage. Thus, employees receive the right of having their personal data “forgotten”.
	Digital rating systems work in real time.	Real-time evaluations of personal services occur only according to established criteria (preferably with the participation of those evaluated).
Human consequences	Digital transformation reduces the proportion of physically stressful task elements.	Physical requirements must be integrated when designing the workflow, for instance via activity-promoting exercise concepts.
	Digital transformation is increasing the proportion of monitoring tasks with a compulsion for constant attention.	Technology design must be such that interaction with the system remains part of the task processing.
	Digital transformation can create work tasks that lead to fatigue-like conditions.	When designing the work, sufficient changes of activity must be provided, e.g., to avoid monotony (psychological requirements).
Holistic work design	The digital transformation harbors the danger of digital Taylorism.	The holistic nature of a work activity must also be a central criterion in the digital age when deciding on the division of labor in the production and service process.
	In the digitalized world of work, technology developers play a key role in determining the division of labor between humans and machines.	Ensuring holistic work tasks needs to begin during the design or development of a digital technology and it must also guide decisions and actions during its implementation and evaluation in the work process.

* Since these are translations, there may be slight shifts in meaning from the original wording.

**Table A2:** Agreement with the theses in the web-based Delphi (1 = not agree at all to 6 = fully agree).

Theses (Abbreviated)	Number of Ratings	Mean	Median	Skew	Std. Error Skew	Sig. Left-Skewed?
Increasing risk of information overload	62	4.55	5.00	−0.75	0.30	Y
Increasing workload	61	4.08	4.00	−0.16	0.31	
Polarization of tasks’ scope	55	3.91	4.00	−0.21	0.32	
Restrictions of temporal freedom	53	2.92	3.00	0.20	0.33	
Increased scope for personal responsibility	55	4.65	5.00	−1.18	0.32	Y
Risk of attack or manipulation of digitally systems	55	5.49	6.00	−1.38	0.32	Y
Systems making decisions without human in the loop	53	4.04	4.00	−0.28	0.33	
Error-proneness of networked technical systems	53	4.23	4.00	−0.22	0.33	
Increase in communication via digital media	55	4.76	5.00	−1.20	0.32	Y
Social support and collegial exchange remain important	58	5.76	6.00	−2.82	0.31	Y
Insufficient consideration of human knowledge	50	2.76	3.00	0.40	0.34	
Use of technologies tailored to technical possibilities	52	4.83	5.00	−1.50	0.33	Y
A new image of man is required	44	2.48	2.00	0.56	0.36	
Low recognizability of algorithmically shaped interaction	52	4.40	5.00	−0.64	0.33	
Option to capture and process a wide range of information	54	5.78	6.00	−3.13	0.33	Y
Faster decisions through algorithms	51	5.27	6.00	−2.47	0.33	Y
High dependence of algorithmic decisions on data quality	49	5.18	6.00	−1.49	0.34	Y
Increase in flat hierarchies and agile working	48	3.13	3.00	−0.12	0.34	
Rise in diverse new forms of employment	51	5.04	5.00	−0.52	0.33	
Rising importance of remote management, virtual teams	52	5.08	5.00	−1.05	0.33	Y
Better ways to address individual needs in work design	51	4.39	4.00	−0.42	0.33	
Differences to keep pace with technological progress	53	5.06	6.00	−1.25	0.33	Y
More learning experiences for a wide range of employees	48	4.31	5.00	−0.62	0.34	
More small-scale work instructions (re-Taylorization)	47	3.51	4.00	−0.23	0.35	
“Change” as the new norm due to innovation cycles	48	4.29	4.00	−0.67	0.34	
Risk of unlimited working hours and constant availability	54	5.09	5.00	−1.59	0.33	Y
Invisibility of mobile workers for coworkers and superiors	50	4.70	5.00	−1.05	0.34	Y
Blurring boundaries between work and private life	53	4.70	5.00	−1.02	0.33	Y
Problem of faulty technical systems without responsibility	50	4.26	5.00	−0.66	0.34	Y
Increase in digital rating systems in the world of work	46	4.52	4.50	−0.57	0.35	
Digital rating systems work in real time	41	4.32	5.00	−0.94	0.37	Y
Decrease in the amount of physically demanding tasks	50	4.00	4.00	−0.45	0.34	
Increased monitoring tasks requiring steady attention	47	3.94	4.00	−0.46	0.35	
Creation of work tasks that lead to fatigue-like conditions	47	4.49	5.00	−0.99	0.35	Y
Inherent danger of digital Taylorism	42	4.24	4.00	−0.95	0.37	Y
Key role of technology developers for the division of labor	48	4.63	5.00	−1.08	0.34	Y

**Table A3:** Ratings on the relevance of the guidelines in the web-based Delphi (1 = totally irrelevant to 6 = very relevant).

Design Guidelines (Abbreviated)	Number of Ratings	Mean	Median	Skew	Std. Error Skew	Sig. Left-Skewed?
Amount and complexity of information manageable by humans	62	5.00	5.00	−1.54	0.30	Y
Balanced ratio between workload and working time	60	4.80	5.00	−1.10	0.31	Y
Preserve and expand the degree of freedom in task processing	51	4.76	5.00	−1.29	0.33	Y
Human determinability of the system support extent.	55	4.31	5.00	−0.76	0.32	Y
Limitation of personal and exercise of organizational responsibility	51	4.84	5.00	−1.07	0.33	Y
Reliability and protection of technologies used against tampering	55	5.27	6.00	−1.34	0.32	Y
Checkability of algorithmic decisions by qualified personnel	55	4.96	5.00	−1.07	0.32	Y
Availability of work-supporting technologies without breakdowns	54	5.07	5.00	−1.53	0.33	Y
Digital work aligned with non-digitally mediated communication	56	4.38	5.00	−0.58	0.32	
Digital work promoting collegial exchange and social support	56	4.39	5.00	−0.62	0.32	
Technical innovations harnessing human experiential knowledge	52	4.60	5.00	−0.88	0.33	Y
Use of technical innovations is evaluated based on humane criteria	53	5.32	6.00	−0.66	0.33	Y
Consideration of human learning ability as a significant value	45	4.29	5.00	−0.70	0.35	Y
User-recognizable autonomous system interaction and results	53	4.77	5.00	−0.83	0.33	Y
Transparent data use and the decision making of technical systems	52	4.58	5.00	−0.75	0.33	Y
Human decision-making authority over a technical system	49	4.88	5.00	−1.67	0.34	Y
Plausibility and fairness of system data verifiable	49	4.80	5.00	−0.93	0.34	Y
Visible employer responsibility for OSH in agile structures	53	5.57	6.00	−1.66	0.33	Y
Realization of OSH in new diverse forms of work and employment	50	5.54	6.00	−2.24	0.34	Y
Responsibility for care in work flexible in time and space	52	5.50	6.00	−0.81	0.33	Y
Digital technologies individually adaptable by the user	48	4.69	5.00	−0.88	0.34	Y
Work support for the disadvantaged and disabled via digital tools	50	4.96	5.00	−1.71	0.34	Y
Raising skills and employability of disabled workers via technology	49	5.08	5.00	−0.86	0.34	Y
Use of technology that preserves and promotes learning at work	48	5.02	5.00	−0.80	0.34	Y
Taking work-integrated learning into account when recruiting	45	4.76	5.00	−0.39	0.35	
Limited working hours and accessibility for work purposes	53	5.45	6.00	−1.44	0.33	Y
Counteracting motivated self-endangerment in mobile work	50	4.80	5.00	−1.08	0.34	Y
Use digital communication for work–life balance	51	5.16	5.00	−1.83	0.33	Y
Unambiguous allocation of decision ownership based technology	51	5.08	5.00	−1.55	0.33	Y
Anonymization of personal data in the medium run	42	4.05	4.00	−0.37	0.37	
Real-time evaluations of personal services only by defined criteria	41	4.37	5.00	−0.88	0.37	Y
Integration of physical demands into the work process	47	4.68	5.00	−0.63	0.35	
Interaction with the system remains part of the task processing	43	4.53	5.00	−0.99	0.36	Y
The design of the work provides sufficient variety of activities	48	5.15	5.00	−1.19	0.34	Y
The holistic nature of a task remaining central to task allocation	47	5.09	5.00	−0.81	0.35	Y
Ensuring holistic work tasks in the design of a digital technology	48	4.92	5.00	−1.05	0.34	Y

**Table A4:** Final coding scheme of the content analysis with coded exemplary comments *.

Main Code	Subcode Level 1	Subcode Level 2	Example Comment	Delphi Content
Evaluation of presented contents	Important: Emphasis on the special relevance, the importance		Transparent AI is important, both for the acceptance of users and for the plannability and traceability of developers.	The decision-making of machine algorithms (AI) in technical processes must be safe and verifiable in all functional areas by suitable specialist personnel.
		Opportunities of digitalization for work design are addressed	Yes, of course—in addition, digitization also opens up the possibility of responding to individual needs. Employers should also take this into account	Even in agile organizational structures and processes and flat hierarchies, employers must take responsibility for the safety and health of employees and communicate this clearly.
	Irrelevant: should not be considered further		Why does this have to be recognizable? What difference does it make to the user?	Interaction with, as well as the result of, autonomous systems must be immediately recognizable to users.
	New: Influence of digitalization shows in a special way		Again, tech systems could help foster collegiality, sharing, and social support.	Digital work must promote collegial exchange and social support.
	Already known/nothing new described		This is old, no news value.	Work support technology should be available without disruption.
		Not specific to digitization	I think this statement is important, but I don’t see why this should be special in the digitization context.	Individual responsibility in the processing of tasks needs limits; organizational responsibility must occur in an appropriate way.
	Inapplicable: inappropriate context shown		I don’t think digital working has to have this goal. I rather think that it is generally important to design work in a way that promotes collegial exchange and social support.	Digital work must promote collegial exchange and social support.
	Unclear/ambivalent: difficult to assess, direction of developments difficult to estimate		Individually set limits are of particular relevance. Considered period of working time too unclear.	Digital communication should promote work-life balance—without extending working hours.
		Opposite direction of action: as the represented	No. When developing a technology, this must first be left out of the equation, since technology is not necessarily developed for a specific work task. Only in connection with the concrete application context can a guarantee of holistic work tasks take place. [...]	Ensuring holistic work tasks needs to begin during the design or development of a digital technology and it must also guide decisions and actions during its implementation and evaluation in the work process.
		Activity/individuality: influence the effects differently	Limitations should be negotiated individually	Working hours and accessibility must be limited.
		Limited agreement: to parts of the thesis/design note.	However, the protection must not lead to the technology application then not becoming more complicated/cumbersome for the user or being associated with a higher susceptibility to malfunctions.	The use of digital technologies must have a reliability appropriate to the application, protected against manipulation at all times, especially by third parties.
	Utopia difficult to implement		This is logical, but seems unrealistic.	Work support technology should be available without disruption.
Missing so far			In my opinion, the perspective of a potentially too high scope of activities is missing here.	Degrees of freedom in task processing should be preserved and, where possible and beneficial, expanded through system design.
	Control/monitoring: as a problem to be considered		[...] It is also important that this does not lead to a form of surveillance that endangers data protection and (mental) health.	Functions of technical systems must be transparent to the user at all times with regard to data use and decision-making.
Argument/reason/example			I tend to agree with this. However, it must also be taken into account that in the initial or familiarization phases, many employees can develop a negative attitude toward new technical solutions that levels out over time. [...].	The degree of system support must be determinable by the human.
Literature/projects/relevant research on the topic			Has been true for many years and continues to have great significance (Ironies of Automation, out-of-the-loop phenomenon).	Digital transformation is increasing the proportion of monitoring tasks with a compulsion for constant attention.
Society/company: social and company aspects are emphasized			[...] In terms of sustainability, however, the requirement should be reconsidered, since in my opinion digital communication is the key to avoiding unnecessary business travel. [...]	Digital work must adhere to opportunities for direct and non-digitally mediated communication.
	Invisibility: references to people not considered in the work system		[...] Solutions are also needed for precarious activities, e.g., in areas that come under strong price and competitive pressure due to the platform economy and/or are currently not regulated by formal self-employment.	The ratio of work quantity and working time must balance each other properly. If the work intensity is high, correspondingly more recovery time should be available.
Ethics/values/meaning: significant			Interactions between system and man are constantly being renegotiated. It is likely that people will fall behind. Especially then, questions of ethics and meaning will gain in importance.	The degree of system support must be determinable by the human.
Linkage other criterion: reference to other thematic assignment			Doesn’t this statement belong more to the human-related consequences?	Users can adapt digital technologies themselves inter- and intra-individually; the competencies required for this must be ensured in order to achieve positive, health-related effects.
	Transparency: Linking with the topic of transparency		However, the machine algorithms must in particular be transparent and comply with values (e.g., the European precautionary principle)	The decision-making of machine algorithms (AI) in technical processes must be safe and verifiable in all functional areas by suitable specialist personnel.
	Dissolution of boundaries: linking with the topic of dissolution of boundaries		The outsourcing of employer duties is not desirable (interested self-endangerment and dissolution of boundaries). Subjectivization of work and (too) high autonomy always bear the risk of endangering health.	Even in agile organizational structures and processes and flat hierarchies, employers must take responsibility for the safety and health of employees and communicate this clearly.
Formulation: Rewording/clarification is proposed	Not future, but present: systems already in use, effects already observable		...that also applies now.	Assuming technical transparency, the assignment and communication of process and decision-making responsibility must be unambiguous.
	Lack of work relevance: of the digitization effects described		Relation of this thesis to the world of work?	Digital technologies should come into play with the support of disadvantaged and low-ability people in mind; no groups of people should experience exclusion.
	Two statements: division desired, only one of the two statements is agreed to		I would fully underline the first sentence. I find the second sentence especially in relation to the first sentence misleading [...].	The ratio of work quantity and working time must balance each other properly. If the work intensity is high, correspondingly more recovery time should be available.
	Lack of depth: wording too trivial, too few facets considered		Very vague wording. It remains unclear what these measures could be.	In order to counteract interested self-endangerment, measures are necessary to make the stress of colleagues working on the move visible.
		Discriminatory power: of the terms/formulation required	See also DSGVO and BDSG for valid formulations related to data protection.	Algorithms convert personal data into an anonymized form for medium-term storage. Thus, employees receive the right of having their personal data “forgotten”.
	New formulation example: or hints on words to be replaced		“The responsibility for the safety and health of employees lies with the employer. Particularly in agile structures/processes, care must be taken to ensure that this can be sensibly exercised and that everyone involved is aware of the responsibility.”	Even in agile organizational structures and processes and flat hierarchies, employers must take responsibility for the safety and health of employees and communicate this clearly.
	Concretization desired		How is “safe”, “suitable specialist personnel” and “all functional areas” defined? [...] Apart from these vague formulations, the statement is to be agreed with.	The decision-making of machine algorithms (AI) in technical processes must be safe and verifiable in all functional areas by suitable specialist personnel.
	Thesis and statement do not fit together		In my opinion, the statement does not fit the thesis. What then is the design proposal regarding blurred boundaries? [...]	Digital communication should promote work-life balance—without extending working hours.
Process-related comments on the course of the Delphi			Apart from that, I thought it was good to be involved in the process, but it was very time-consuming. [...]	Do you find the developed criteria important and suitable? Does the designation seem appropriate to you or do you have alternative suggestions?

* Since these are translations, there may be slight shifts in meaning from the original wording.
